# Liposuction for Advanced Lymphedema: A Multidisciplinary Approach for Complete Reduction of Arm and Leg Swelling

**DOI:** 10.1245/s10434-015-4700-3

**Published:** 2015-06-30

**Authors:** John Boyages, Katrina Kastanias, Louise A. Koelmeyer, Caleb J. Winch, Thomas C. Lam, Kerry A. Sherman, David Alex Munnoch, Håkan Brorson, Quan D. Ngo, Asha Heydon-White, John S. Magnussen, Helen Mackie

**Affiliations:** Department of Clinical Medicine, Faculty of Medicine and Health Sciences, 2 Technology Place, Macquarie University, Sydney, NSW Australia; Genesis Cancer Care, Macquarie University Hospital, 3 Technology Place, Macquarie University, Sydney, NSW Australia; Centre for Emotional Health, Department of Psychology, Macquarie University, Sydney, NSW Australia; Department of Plastic Surgery, Ninewells Hospital, Dundee, UK; Plastic and Reconstructive Surgery, Department of Clinical Sciences, Skåne University Hospital, Lund University, Malmö, Sweden; The Clinic Physiotherapy, Macquarie University Hospital, 2 Technology Place, Macquarie University, Sydney, NSW Australia; Macquarie Medical Imaging, Macquarie University Hospital, 3 Technology Place, Macquarie University, Sydney, NSW Australia; Mt. Wilga Rehabilitation Hospital, Hornsby, NSW Australia

## Abstract

**Purpose:**

This research describes and evaluates a liposuction surgery and multidisciplinary rehabilitation approach for advanced lymphedema of the upper and lower extremities.

**Methods:**

A prospective clinical study was conducted at an Advanced Lymphedema Assessment Clinic (ALAC) comprised of specialists in plastic surgery, rehabilitation, imaging, oncology, and allied health, at Macquarie University, Australia. Between May 2012 and 31 May 2014, a total of 104 patients attended the ALAC. Eligibility criteria for liposuction included (i) unilateral, non-pitting, International Society of Lymphology stage II/III lymphedema; (ii) limb volume difference greater than 25 %; and (iii) previously ineffective conservative therapies. Of 55 eligible patients, 21 underwent liposuction (15 arm, 6 leg) and had at least 3 months postsurgical follow-up (85.7 % cancer-related lymphedema). Liposuction was performed under general anesthesia using a published technique, and compression garments were applied intraoperatively and advised to be worn continuously thereafter. Limb volume differences, bioimpedance spectroscopy (L-Dex), and symptom and functional measurements (using the Patient-Specific Functional Scale) were taken presurgery and 4 weeks postsurgery, and then at 3, 6, 9, and 12 months postsurgery.

**Results:**

Mean presurgical limb volume difference was 45.1 % (arm 44.2 %; leg 47.3 %). This difference reduced to 3.8 % (arm 3.6 %; leg 4.3 %) by 6 months postsurgery, a mean percentage volume reduction of 89.6 % (arm 90.2 %; leg 88.2 %) [*p* < 0.001]. All patients had improved symptoms and function. Bioimpedance spectroscopy showed reduced but ongoing extracellular fluid, consistent with the underlying lymphatic pathology.

**Conclusions:**

Liposuction is a safe and effective option for carefully selected patients with advanced lymphedema. Assessment, treatment, and follow-up by a multidisciplinary team is essential.

“I was always alone and at my lowest point I wanted to have my leg cut off. I remember going to my General Practitioner and asking if that were possible”, first leg liposuction patient (Fig. 1).

**Fig. 1:**
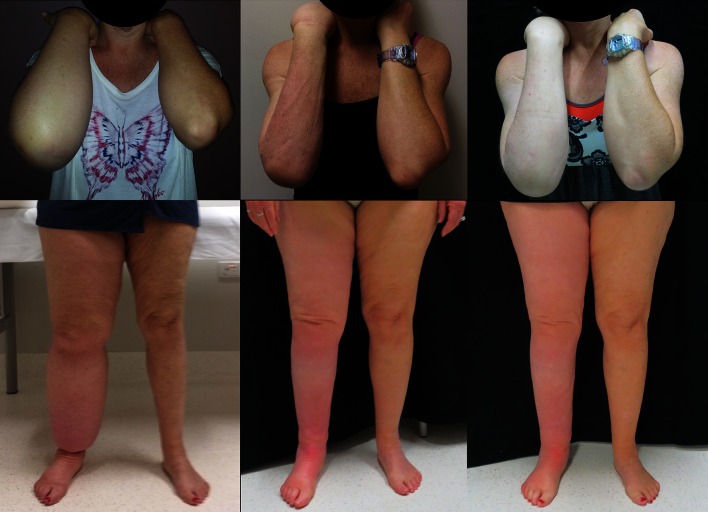
First arm and leg liposuction patients, both right-side affected. *Left panel* pre-liposuction, *middle panel* 6 months post-liposuction, *right panel* 12 months post-liposuction

The negative impact of advanced lymphedema on the physical and emotional health of cancer survivors cannot be understated.^1^–^9^ Symptomatic management, rather than cure, is available for the large proportion of breast (21 %), gynecologic (20 %), melanoma (16 %), and genitourinary (10 %) cancer survivors (among others) who develop lymphedema.^10^,^11^ Early lymphedema swelling can usually be managed with decongestive lymphatic therapy (DLT): lymphatic drainage massage, compression garments, exercises, and skin care.^2^ However, until recently no further treatments existed for advanced lymphedema resistant to DLT. This report presents volume, bioimpedance, functional, and emotional outcomes from the first Australian clinic conducting liposuction for advanced lymphedema.

European teams first developed liposuction protocols for advanced lymphedema,^12^–^14^ the rationale being that swelling in advanced lymphedema is not only due solely to lymphatic fluid but also to accumulating adipose tissue^15^,^16^ and sometimes fibrosis.^17^ Liposuction significantly reduces excess limb volume^12^,^17^,^18^ for patients with advanced arm or leg lymphedema,^12^,^13^,^19^,^20^ with ongoing reduction maintained by continuous compression garment use.^17^,^18^,^21^ Furthermore, Swedish data suggest that liposuction reduces episodes of cellulitis (often requiring hospitalization) from an annual incidence of 40 % preoperatively to 10 % postoperatively.^22^ The best outcomes post-liposuction are achieved by patients who return frequently for monitoring and garment renewal.^14^,^18^ However, this very need for ongoing monitoring and expensive garments raises questions about whether liposuction will generalize out of the universal healthcare contexts of Sweden and the UK to countries such as Australia and the US where the cost of lymphedema treatment is paid for by the individual.^23^ Furthermore, temperatures in Sweden and the UK are comparatively low, making it more comfortable to wear compression garments. Nevertheless, liposuction remains the best contemporary surgical option to reduce swelling for patients with advanced lymphedema because alternatives, such as lymph node transfer (LNT) microsurgery,^7^,^24^–^26^ focus only on restoring lymphatic function in the affected area. Although perhaps stopping the progression of advanced lymphedema, such techniques cannot foreseeably reduce the fatty and fibrotic swelling already present.

Two additional topics are not adequately addressed in liposuction research. First, although liposuction substantially improves function,^3^ studies disagree as to whether liposuction improves emotional outcomes.^3^,^13^ Second, no research has measured extracellular lymphatic fluid pre- and post-liposuction using bioimpedance, an assessment that will soon become standard-of-care for lymphedema.^27^ Therefore, in 2012, clinicians and researchers at Macquarie University convened a multidisciplinary Advanced Lymphedema Assessment Clinic (ALAC) for privately funded patients to carefully implement and evaluate liposuction for lymphedema. This report presents volume, bioimpedance, functional, and emotional outcomes after 2 years of this program (median postsurgery follow-up, 9 months; range 3–18).

## Methods

### Setting

Specialists in rehabilitation (HM), plastic surgery (TL, QN), imaging (JM), oncology (JB), and allied health (LK, AH-W) established ALAC in May 2012. TL was trained in surgical technique by AM. TL, HM, and LK received further instruction in technique and follow-up protocol from HB in Sweden. HB also provided analysis tools to ensure comparability with previous studies. At ALAC, a rehabilitation specialist with expertise in lymphedema (HM) took a patient’s medical history and assessed their eligibility for surgery. A lymphedema-trained physiotherapist (AH-W) measured limb volumes, took bioimpedance spectroscopy (L-Dex), and assessed function. Eligible patients discussed liposuction with the plastic surgeon (TL). Preoperative magnetic resonance imaging (MRI) assessed the location and extent of lymphatic fluid and fat for surgical planning. If there was evidence of pitting edema or substantial lymphatic fluid on MRI, patients completed modified intensive DLT consisting of 1–2 weeks of presurgery bandaging at a private rehabilitation hospital. Patients returned to ALAC 2–6 weeks postsurgery and at 3, 6, 9, and 12 months to measure limb volume and order new compression garments, and for reviews 6-monthly thereafter. If progress was good and new garments were not required, 3- and/or 9-month assessment was omitted. MRI was repeated at 6 months to reassess fluid and fat distribution, and will be analyzed in future research to improve muscle, fat, and fluid differentiation and measurement. All cases were reviewed at monthly multidisciplinary team meetings.

Patients’ motivation to wear, and their ability to pay for, continuous garments was assessed. Patients without private health insurance self-funded a 5-day hospital stay, theater room hire, physiotherapy, and inpatient DLT.

### Patients and Eligibility

Between May 2012 and May 2014, a total of 104 patients were assessed. Eligibility criteria for liposuction were (i) unilateral nonpitting primary or secondary advanced International Society of Lymphology (ISL) stage II or III lymphedema;^12^,^13^ (ii) limb volume difference greater than 25 % (calculated using the truncated-cone method);^12^,^13^ and (iii) DLT provided no further volume reductions. Patients were excluded if they had not undertaken maximum DLT (*n* = 3), had active recurrent cancer (*n* = 2), bilateral lymphedema (*n* = 5), frailty (*n* = 3), or were reluctant to wear compression garments continuously (*n* = 5).^17^,^18^,^21^

By May 2014, 21 of 55 eligible patients underwent liposuction and were at least 3 months postsurgery. An additional seven patients underwent surgery (four did not consent to research and three had less than 3 months of follow-up), eight were booked in, and 19 patients decided against surgery or found the costs prohibitive (22 %).

### Measures

#### Limb Volume

Volume was calculated using 4-cm truncated cone circumferential measurements.^28^ A measuring board was used. Arms were measured seated, with the arm in horizontal abduction, hand pronated, and commencing at the ulnar styloid. Leg measurements were taken in the supine position with legs slightly abducted, commencing at the ankle between the lateral and medial malleolus. Individual limb volume, difference between limbs, and percentage difference were calculated, comparing the affected limb with the unaffected limb.^28^,^29^

#### Bioimpedance Spectroscopy (L-Dex)

Measurements were taken supine using the ImpediMed L-Dex^®^ machine (ImpediMed, Carlsbad, CA, USA) that assesses extracellular fluid in a unilateral limb using a low-voltage electrical current. L-Dex readings are an impedance ratio comparing the unaffected limb with the affected limb, with the unaffected limb acting as a patient-specific internal control.^30^ Skin was cleaned with alcohol swabs, and electrodes placed according to the manufacturer’s recommendations. Normal (no lymphedema) L-Dex readings ranged between −10 and 10.^31^

#### Function/Emotions

Functional impairment was assessed using the Patient-Specific Functional Scale (PSFS).^32^ The PSFS is reliable and valid across contexts,^33^ and sensitive to change in breast cancer survivors,^34^ but not previously validated for lymphedema. Patients listed three personally important activities impaired by lymphedema (e.g. ‘brushing my hair’), then rated the extent to which lymphedema impaired each activity (‘0’, not able to perform, to ‘10’, able). Therefore, although the specific activities nominated by individuals differed substantially, impairment ratings were standardized and thus comparable across activities, patients, and time.^32^ Impairment ratings were summed for each patient, forming an individualized index of functional impairment (range 0–30), with higher numbers indicating less impairment. It was not appropriate to calculate internal consistency (e.g. Cronbach’s *α*) because activities were idiosyncratic across patients.

To complement the PSFS, patients also rated the impact of lymphedema on six functional/emotional domains drawn from previous research^35^,^36^ (see the Appendix): pain, heaviness, extent of swelling, degree of self-consciousness, anxiety, and negative emotions (‘0’, not at all, to ‘10’, extremely so). Although the domains were correlated (Cronbach’s *α* = 0.76), they were reported individually because they reflect diverse content.

### Surgical Technique and Compression Therapy

The surgical procedure was identical to that described in Sweden.^12^,^13^,^19^,^20^ Liposuction was performed under general anesthesia following limb exsanguination and tourniquet application. Using specialized Helixed Tri Port III cannulas (22 and 30 cm long, 4–5 mm wide) connected to a vacuum pump, subcutaneous tissue was removed through multiple small incisions along the limb. Presurgical limb volume determined how much tissue to remove to equalize volume relative to the unaffected limb. Compression garments were applied to the affected limb immediately postsurgery prior to tourniquet release—custom-made 30 mmHg JOBST^®^ Elvarex^®^ for arms, or Ready Wraps (Solaris) for legs. From 1-week postsurgery, all leg patients wore JOBST^®^ Elvarex^®^ custom-made compression garments 50–80 mmHg. Initial postsurgical garments were measured using the circumference of the unaffected limb; subsequent measurements were obtained from the operated limb by a trained garment fitter. Every order consisted of two garments, allowing one to be worn while the other was washed. Throughout follow-up, compression garments alone were used in areas where liposuction was performed. However, DLT was used, where indicated, in areas where liposuction was not performed (hands or feet) or areas that cannot be adequately compressed (shoulder or hip). These areas are not included in limb volume measurements.^28^,^29^

### Statistical Analysis

Volume, L-Dex, and function were compared with paired samples *t* tests. As analysis entailed five pairwise comparisons between succeeding timepoints (pre vs. post, pre vs. 3-month, etc.), the Bonferroni correction was applied, (i.e. *p* value was considered significant at 0.05/5 = 0.01^37^). With 21 participants, we calculated 80 % power at *p* = 0.01 to detect a *d* = 0.8 decrease in limb volume and L-Dex. However, as fewer participants had longer follow-up, less power was available at 12-month follow-up. Hence, further clinically meaningful volume reductions may not attain statistical significance. Statistical tests were performed using SPSS version 21 for Windows (IBM Corporation, Armonk, NY, USA). Data were collected with patients’ consent and Macquarie University Human Research Ethics Committee approval.

## Results

Table 1 details characteristics of 15 arm and 6 leg liposuction patients. Cancer-related secondary lymphedema was a more common reason for liposuction (85.7 %) than primary (congenital) lymphedema (14.3 %), with breast cancer treatment being the most common underling cause (66.7 %). Patients with arm lymphedema were older (mean_(arm)_ 57.8 years, range 25–69; mean_(leg)_ 50.7, range 18–66) but had less longstanding lymphedema (mean_(arm)_ 9.1 years, range 2–29), than patients with leg lymphedema (mean_(leg)_ 15.5, range 3–42). All patients were female, reflecting the sex disparity in lymphedema prevalence due to breast cancer.

**Table 1:** Participant characteristics

	Upper Limb	Lower Limb	Total	(%)
	*n*	*n*		
Sex				
Female	15	6	21	100
Age (years)				
<50	2	1	3	14.3
≥50	13	5	18	85.7
Mean (SD)	57.8 (12.2)	50.7 (16.9)		
Range	25–69	18–66		
BMI (kg/m^2^)				
Mean (SD)	28.3 (4.1)	29.69 (2.7)		
Low to normal (<25)	3	0	3	14.3
Overweight (25–30)	7	4	11	52.4
Obese (>30)	5	2	7	33.3
Cancer diagnosis				
Breast	14	0	14	66.7
Gynecological	0	4	4	19.0
Non-cancer	1	2	3	14.3
Nodal surgery				
Nil	1	5	6	28.6
Sentinel node biopsy	1	0	1	4.8
Nodal dissection	13	1	14	66.7
Radiotherapy				
Yes	11	1	12	57.1
No	4	5	9	42.9
Chemotherapy				
Yes	11	2	13	61.9
No	4	4	8	38.1

*SD* standard deviation, *BMI* body mass index

Significant post-liposuction reduction in limb volume was achieved for all patients (Table 2). Mean preoperative limb difference was 45.1 % (range 23–83), decreasing between 2 and 6 weeks postsurgery to 13.2 % (range −2 to 24), a significant 68.2 % reduction (range 35–104, *t*(20) = 9.66; *p* < 0.001). Limb volume difference further reduced to 3.8 % by 6 months postsurgery, an 89.6 % (range 38–149) reduction of presurgical volume (*t*(18) = 9.17; *p* < 0.001). This near-complete reduction was maintained to 12 months (*n* = 8), a 97.7 % reduction (range 73–123, *t*(8) = 5.73; *p* < 0.001).

**Table 2:** Excess volume and L-Dex value pre- and postoperatively

	Preoperative	6 months	12 months	18 months
Upper limb (*n*)	15	12	7	1
L-Dex [mean (range)]	41.2 (18–75)	35.3 (14–49)	25.1 (13–45)	27
Significance	–	*p* = 0.068	*p* = 0.018	–
Mean excess volume [ml (range)]	1139.5 (645–1755)	67.9 (−697 to 422)	18.7 (−244 to 218)	−339^a^
Mean excess volume [% (range)]	44.2 (27–67)	3.6 (−21 to 21)	1.3 (−5 to 8)	−11^a^
Significance	–	*p* *<* 0.001	*p* = 0.001	–
Lower limb (*n*)	6	5	1	0
L-Dex [mean (range)]	46.9 (12–97)	49.3 (33–71)	39.0	–
Significance	–	*p* = 0.746	–	–
Mean excess volume [ml (range)]	4058 (2068–8294)	400 (−112 to 867)	−103	–
Mean excess volume [% (range)]	47.3 (23–83)	4.3 (−1 to 11)	−1.0	–
Significance	–	*p* = 0.018	–	–

Significance assessed using paired samples *t* tests compared with preoperative value

^a^This patient gained weight overall but increased in fat volume in the unaffected arm only, i.e. the affected arm is now smaller than the unaffected arm

Mean preoperative L-Dex was 42.9 (range 12–97) for all patients. L-Dex increased 4 weeks postsurgery to 55.0 (range 32–73), reflecting the extracellular fluid associated with postsurgical swelling (*t*(18) = −2.51; *p* = 0.02). L-Dex was at presurgical values 6 months postsurgery (mean 38.1, range 14–71, *t*(14) = 1.68; *p* = 0.12) and reduced below presurgical values at 12 months (mean 27.1, range 13–45, *t*(7) = 3.38; *p* = 0.02). However, L-Dex values remained elevated above the ‘normal’ range (0 ± 10), likely indicating ongoing lymphatic pathology. Although these comparisons were not statistically significant (applying the Bonferroni correction), change from timepoint to timepoint always exceeded the 10 points considered clinically significant for L-Dex.

Functionally, all patients reported improvements on the PSFS index of personally important activities (Table 3) by 6 months postsurgery (*p* < 0.01). Improvements were also evident in the standardized domains of pain, heaviness, self-consciousness, levels of anxiety, perceived degree of swelling, and emotional impact; such improvements were statistically significant, with the exception of pain in the lower limb and anxiety about the upper limb. There have been no surgical complications; one patient had poor compression garment compliance.

**Table 3:** Functional and emotional impact of lymphedema before and after liposuction

	Preoperative	6 months	Effect at 6-month follow-up		
	Mean (range)	Mean (range)	*N*	*t*	*p* value
PSFS functional impairment^a^					
Upper limb	11.1 (4–21)	22.1 (9–30)	7	3.86	0.008
Lower limb	7.4 (4–9)	28.0 (27–29)	5	23.6	<0.001
Pain^b^					
Upper limb	3.9 (0–8)	0.8 (0–3)	9	3.60	0.007
Lower limb	3.7 (0–8)	0.2 (0–1)	5	2.50	0.07
Heaviness^b^					
Upper limb	6.7 (3–10)	0.3 (0–2)	9	9.71	<0.001
Lower limb	8.2 (6–10)	0.4 (0–2)	5	7.65	0.002
Self-consciousness^b^					
Upper limb	6.9 (2–10)	0.6 (0–3)	9	5.94	<0.001
Lower limb	8.2 (4–10)	0	5	24.59	<0.001
Anxious^b^					
Upper limb	5.1 (0–10)	0.2 (0–2)	9	3.31	0.11
Lower limb	7.2 (5–10)	0	5	9.36	<0.001
Swollen^b^					
Upper limb	6.9 (2–10)	1.8 (0–4)	9	5.49	<0.001
Lower limb	9.0 (8–10)	1.6 (0–2)	5	9.89	<0.001
Impact on emotions^b^					
Upper limb	6.0 (0–10)	1.0 (0–4)	9	4.07	0.004
Lower limb	7.8 (2–10)	0.6 (0–3)	5	12.37	<0.001

Significance assessed using paired samples *t* tests compared with preoperative value

*PSFS* Patient-Specific Functional Scale^32^

^a^Scores ranged from ‘0’ (not able to perform three activities at all) to ‘30’ (able to perform three activities perfectly)

^b^Scores ranged from ‘0’ (not at all) to ‘10’ (extremely so)

## Discussion

“The most emotional day was when I got my first garment on – it was mind blowing to see my ankle and calf so much the same size as my other leg. Everything in my life has now changed – for the better”, first leg liposuction patient (Fig. 1).

A multidisciplinary team convened Australia’s first clinic (ALAC) providing liposuction for lymphedema. European protocols^12^,^14^,^18^,^20^,^21^ were applied within a unique multidisciplinary context, where the surgeon and occupational therapist were joined by an oncologist and physiotherapist, and led by a rehabilitation specialist. ALAC conducted surgery on cancer-related lymphedema and primary (congenital) lymphedema. Liposuction was effective in this privately-funded context, eliminating excess volume on average and improving symptoms and function in the affected limb. Patients maintained reductions to 12 months by continuously wearing compression garments.

These results are comparable with international standards. Arm liposuction achieved mean volume reductions of 90 % at 6 months and 97 % at 12 months postsurgery compared with 103 and 111 % in Sweden,^38^ and 92 and 101 % (range 69–148) in the UK.^13^ Results 12 months postsurgery were 116 % (range 75–233) in The Netherlands^14^ and 111 % (range 90–130) in the US.^24^ Leg surgery achieved volume reductions of 88 % at 6 months and 101 % (one patient) at 12 months postsurgery compared with 84 % at 3 months and 105 % at 12 months postsurgery in Sweden,^20^ and 86 % (range 81–97) 12 months postsurgery in the US.^24^

Previous liposuction research demonstrated improved quality of life^3^ and overall wellbeing,^13^ but disagreed about improvements in emotional wellbeing.^3^,^13^ In ALAC, patients reported substantial and statistically significant reduction in lymphedema impact on important activities, improved limb function, and reduced lymphedema-specific emotional distress. Thus, liposuction has substantial functional and psychological benefit.

These results are preliminary owing to the small number of patients. Nevertheless, they are clinically and statistically significant despite the expense associated with self-funding compression garments and the discomfort of wearing garments continuously in Australia’s hot climate. Liposuction addresses physical swelling but not the underlying lymphatic dysfunction; therefore, patients must maintain garment use to continue to benefit from the surgery. However, we have anecdotes that some patients are wearing compression intermittently yet continuing to maintain reduction. ALAC is planning to explore whether less burdensome garment requirements are possible through a randomized controlled trial of durations and levels of compression after liposuction for upper limb lymphedema.

All patients experienced benefit and there were no adverse events. This is consistent with lymphoscintigraphy studies demonstrating that liposuction is not associated with further damage to lymphatic transport in arms with lymphedema,^39^ and with a cadaveric study which demonstrated that longitudinal liposuction does not damage the epifascial lymph vessels.^40^ These results are early indications that liposuction is safe and will not additionally compromise lymphatic drainage, either in primary or secondary lymphedema.

Although the treatment protocol at ALAC was similar to others,^12^,^14^,^18^,^20^,^21^ the clinic composition was expanded. In addition to a surgeon and occupational therapist/physiotherapist, the lead clinician was a rehabilitative specialist, i.e. liposuction was seen as the first step in ongoing nonsurgical management, including compression garment use requiring regular reordering.^13^,^21^,^24^ In addition, an oncologist was present to balance cosmesis, function, and quality of life against prognosis, if necessary. ALAC holds the conviction that the highest standard of care is achieved within this multidisciplinary environment.

Future research should determine selection criteria and sequencing for other surgical procedures. Our clinic is evaluating LNT^7^,^41^ for patients with ISL stage I or II lymphedema where pitting edema rather than fat is the clinical presentation. LNT+ delayed autologous breast reconstruction is another potential approach for patients undergoing a mastectomy.^24^–^26^ Furthermore, surgeries might be combined, such as liposuction to remove fatty lymphedema followed by LNT± autologous breast reconstruction to eliminate the need for compression garments. The appropriate surgical approach can be defined using lymphedema clinical characteristics;^24^ ALAC holds the additional belief that patient factors such as motivation and the ability to pay for compression garments must be assessed.

## Conclusions

With continued compression garment use, ALAC expects patients to maintain limb reductions as reported in 5- to 15-year follow-up in Europe.^13^,^20^,^38^ Only one patient has failed to maintain compliance for garment use, for unknown reasons. However, these Australian patients were carefully selected with regard to their long-term ability to pay for garments. Costs prevented 12 eligible patients from undergoing surgery. Considering the significant functional gains observed in this and other studies,^3^,^13^,^24^ and reduced hospitalizations due to infection,^8^,^9^,^22^ governments and insurance companies should consider the economic value of funding liposuction for advanced lymphedema.

## References

[1] Fu MR, Ridner SH, Hu SH, Stewart BR, Cormier JN, Armer JM (2013). Psychosocial impact of lymphedema: a systematic review of literature from 2004 to 2011. Psychooncology.

[2] Lymphoedema Framework (2006). Best practice for the management of lymphoedema. International Consensus Document.

[3] Brorson H, Ohlin K, Olsson G, Langstrom G, Wiklund I, Svensson H (2006). Quality of life following liposuction and conservative treatment of arm lymphedema. Lymphology.

[4] Morgan PA, Franks PJ, Moffatt CJ (2005). Health-related quality of life with lymphoedema: a review of the literature. Int Wound J.

[5] Pusic A, Cemal Y, Albornoz C (2013). Quality of life among breast cancer patients with lymphedema: a systematic review of patient-reported outcome instruments and outcomes. J Cancer Surviv.

[6] Ridner SH (2009). The psycho-social impact of lymphedema. Lymphat Res Biol.

[7] Becker C, Assouad J, Riquet M, Hidden G (2006). Postmastectomy lymphedema: long-term results following microsurgical lymph node transplantation. Ann Surg.

[8] Inghammar M, Rasmussen M, Linder A (2014). Recurrent erysipelas: risk factors and clinical presentation. BMC Infect Dis.

[9] Pavlotsky F, Amrani S, Trau H (2004). Recurrent erysipelas: risk factors. J Dtsch Dermatol Ges.

[10] Cormier JN, Askew RL, Mungovan KS, Xing Y, Ross MI, Armer JM (2010). Lymphedema beyond breast cancer: a systematic review and meta-analysis of cancer-related secondary lymphedema. Cancer.

[11] DiSipio T, Rye S, Newman B, Hayes S (2013). Incidence of unilateral arm lymphoedema after breast cancer: a systematic review and meta-analysis. Lancet Oncol.

[12] Brorson H (2000). Liposuction gives complete reduction of chronic large arm lymphedema after breast cancer. Acta Oncol.

[13] Schaverien MV, Munro KJ, Baker PA, Munnoch DA (2012). Liposuction for chronic lymphoedema of the upper limb: 5 years of experience. J Plast Reconstr Aesthet Surg.

[14] Damstra RJ, Voesten HG, Klinkert P, Brorson H (2009). Circumferential suction-assisted lipectomy for lymphoedema after surgery for breast cancer. Br J Surg.

[15] Brorson H, Ohlin K, Olsson G, Nilsson M (2006). Adipose tissue dominates chronic arm lymphedema following breast cancer: an analysis using volume rendered CT images. Lymphat Res Biol.

[16] Brorson H, Ohlin K, Olsson G, Karlsson MK (2009). Breast cancer-related chronic arm lymphedema is associated with excess adipose and muscle tissue. Lymphat Res Biol.

[17] Brorson H (2012). From lymph to fat: liposuction as a treatment for complete reduction of lymphedema. Int J Low Extrem Wounds.

[18] Brorson H (2010). From lymph to fat: complete reduction of lymphoedema. Phlebology..

[19] Brorson H (2003). Liposuction in arm lymphedema treatment. Scand J Surg.

[20] Brorson H. Liposuction normalizes elephantiasis of the leg: a prospective study with an eight-year follow-up. Presented at the American Association of Plastic Surgeons 91st Annual Meeting; 14–17 Apr 2012; San Francisco.

[21] Brorson H, Svensson H (1998). Liposuction combined with controlled compression therapy reduces arm lymphedema more effectively than controlled compression therapy alone. Plast Reconstr Surg.

[22] Brorson H, Svensson H (1997). Skin blood flow of the lymphedematous arm before and after liposuction. Lymphology.

[23] Australasian Lymphoedema Association. Compression garment schemes. 2014. http://www.lymphoedema.org.au/ALA/Lymphoedema/Compression_Garment_Schemes/ALA/Lymphodema/Compression_Garment_Schemes.aspx?hkey=8fa67649-5312-4ec5-b2ca-5efad3eff02a. Accessed 11 July 2014.

[24] Granzow JW, Soderberg JM, Kaji AH, Dauphine C (2014). An effective system of surgical treatment of lymphedema. Ann Surg Oncol.

[25] Honkonen KM, Visuri MT, Tervala TV (2013). Lymph node transfer and perinodal lymphatic growth factor treatment for lymphedema. Ann Surg.

[26] Saaristo AM, Niemi TS, Viitanen TP, Tervala TV, Hartiala P, Suominen EA (2012). Microvascular breast reconstruction and lymph node transfer for postmastectomy lymphedema patients. Ann Surg.

[27] Ward LC (2009). Is BIS ready for prime time as the gold standard measure?. J Lymphoedema.

[28] Brorson H, Hoijer P (2012). Standardised measurements used to order compression garments can be used to calculate arm volumes to evaluate lymphoedema treatment. J Plast Surg Hand Surg.

[29] Taylor R, Jayasinghe UW, Koelmeyer L, Ung O, Boyages J (2006). Reliability and validity of arm volume measurements for assessment of lymphedema. Phys Ther.

[30] Ward LC, Essex T, Cornish BH (2006). Determination of cole parameters in multiple frequency bioelectrical impedance analysis using only the measurement of impedances. Physiol Meas.

[31] Fu MR, Cleland CM, Guth AA (2013). L-dex ratio in detecting breast cancer-related lymphedema: reliability, sensitivity, and specificity. Lymphology.

[32] Stratford P (1995). Assessing disability and change on individual patients: a report of a patient specific measure. Physiother Can.

[33] Horn KK, Jennings S, Richardson G, Van Vliet D, Hefford C, Abbott JH (2012). The patient-specific functional scale: psychometrics, clinimetrics, and application as a clinical outcome measure. J Orthop Sports Phys Ther.

[34] Campbell KL, Pusic AL, Zucker DS (2012). A prospective model of care for breast cancer rehabilitation: function. Cancer.

[35] Weiss J, Spray B (2002). The effect of complete decongestive therapy on the quality of life of patients with peripheral lymphedema. Lymphology.

[36] Zigmond AS, Snaith RP (1983). The Hospital Anxiety and Depression Scale. Acta Psychiatr Scand.

[37] Howell DC (2012). Statistical methods for psychology.

[38] Brorson H. Liposuction of postmastectomy arm lymphedema completely removes excess volume: a 15 year study. American Association of Plastic Surgeons 90th annual meeting; 9–12 Apr 2011; Boca Raton (FL).

[39] Brorson H, Svensson H, Norrgren K, Thorsson O (1998). Liposuction reduces arm lymphedema without significantly altering the already impaired lymph transport. Lymphology.

[40] Frick A, Hoffmann JN, Baumeister RG, Putz R (1999). Liposuction technique and lymphatic lesions in lower legs: anatomic study to reduce risks. Plast Reconstr Surg.

[41] Granzow JW, Soderberg JM, Kaji AH, Dauphine C (2014). Review of current surgical treatments for lymphedema. Ann Surg Oncol.

